# Active Sites Regulation for High-Performance Oxygen Evolution Reaction Electrocatalysts

**DOI:** 10.3389/fchem.2022.889470

**Published:** 2022-04-27

**Authors:** Yu Tang, Tianyi Zhang, Xuan Wu, Shukang Deng

**Affiliations:** ^1^ Education Ministry Key Laboratory of Renewable Energy Advanced Materials and Manufacturing Technology, Yunnan Normal University, Kunming, China; ^2^ School of Literature and Art Media, Anhui International Studies University, Hefei, China

**Keywords:** oxygen evolution reaction, precious metals, transition metals, oxides, Sulfides

## Abstract

Electrochemical water splitting to produce molecular hydrogen and oxygen provides a promising strategy engineering for scalable hydrogen production with high purity. Unfortunately, the sluggish kinetics of oxygen evolution reactions (OER) due to the interdependence multiple steps procedure require high overpotential to achieve appreciable catalytic current density, resulting in relatively low energy conversion efficiencies. Therefore, development of high-performance OER electrocatalysts is vital to drive the commercial application of water splitting. This review highlights current progress of representative catalyst electrocatalysts in the past decade. Active site regulation for excellent OER performance of precious metal single atoms catalyst, high-entropy alloy, transition metals oxides, transition metal chalcogenide are emphasized. And a more in-depth exploration of OER reaction mechanism by *in situ* technique and DFT results will be conducted. This review can provide the basis for the development and modification of OER electrocatalysts.

## Introduction

Hydrogen energy is regarded as a renewable energy with application prospect because of its advantages such as wide source, high combustion heat value, high efficiency and zero pollution (Cai et al., 2020; Zhao et al., 2020; Bai et al., 2021; Tang et al., 2021). Although hydrogen is abundant in earth, it mainly exists in the natural world in the forms of a compound in water, fossil fuels and other media, and rarely exists in the form of H_2_ (Yan et al., 2021; Yu et al., 2022a). Therefore, the realization of efficient hydrogen production is the basis for the application of hydrogen energy (Yao et al., 2019; Wang et al., 2020; Zhao et al., 2021; Yu et al., 2022b). Electrolysis of water can directly convert water into hydrogen and oxygen to obtain high purity hydrogen. At the same time, the source of water is abundant, so the preparation of hydrogen by electrolysis of water is regarded as an ideal method for hydrogen production (Shah et al., 2022). Electrocatalytic water splitting includes hydrogen evolution reaction (HER) on cathode and oxygen evolution reaction (OER) on anode. However, OER is a four-electron coupled reaction, generating a high kinetic barrier and suffering from high overpotential and low efficiency (Hu et al., 2021). At present, the precious metals Ir, Ru, and their oxides show excellent OER electrocatalytic performance (Lee et al., 2012; Danilovic D. N. et al., 2014; Osgood et al., 2016). However, due to the scarcity of earth content, high price, and poor stability, precious metal catalysts cannot meet the large-scale industrial application of hydrogen production by electrolysis of water. Therefore, to exploit low cost, non-noble metal electrocatalyst and replace noble metal catalyst have become an important research topic, and design and modification of the active site have been recognized as the main approach to promote the intrinsic activity.

In general, the OER is a more complex process in comparison with the HER system, the mechanism of OER is complicated and still debatable on the anode catalysts. For conventional OER mechanism, a four-step proton/electron transfer step was proposed as described in Eqs 1–4.

$${\text{OH}}^{-}+{}^{\text{∗}}\to {{\text{HO}}^{\text{∗}}+\text{e}}^{-}$$ (1)

$${\text{HO}}^{\text{∗}}\to {{\text{O}}^{\text{∗}}+\text{e}}^{-}{+\text{H}}^{+}$$ (2)

$${{\text{O}}^{\text{∗}}+\text{H}}^{+}\to {{\text{HOO}}^{\text{∗}}+\text{e}}^{-}$$ (3)

$${\text{HOO}}^{\text{∗}}\to {{}^{\text{∗}}+\text{O}}_{2}\left(\text{g}\right){+\text{e}}^{-}{+\text{H}}^{+}$$ (4)

where ^∗^ and ^∗^OH, ^∗^O, ^∗^OOH represent the active site and the adsorbed intermediate on the active site, respectively. In detail, OH^−^ groups first adsorb on catalyst surface with release of an electron. Then the adsorbed ^∗^OH undergoes deprotonation to form O^∗^. The following O^∗^to react with another OH^−^ to form the HOO^∗^intermediate subsequently decomposes to O_2_(g) and closing the whole OER cycle. The Gibbs free-energy change (ΔG_i_) for these four consecutive electrochemical reactions (Eqs 1–4) can be described in Eqs 5–8. The proton/electron reaction step with the maximum ΔG is regarded as the determining step, and the overpotential can be represented by ΔG_max_/e-1.23. An ideal OER requires all the four elemental steps with reaction free energies 1.23 eV to realize no overpotential (ΔG_1_ = ΔG_2_ = ΔG_3_ = ΔG_4_ = 1.23 eV). Therefore, modulating reasonably the adsorption energy of intermediates ^∗^OH, ^∗^O, and ^∗^OOH) by optimized electronic structure has been considered as one of the promising strategy for enhancing OER.

$$\Delta G_{1}=\Delta G_{HO*}-\Delta G_{*}+\frac{1}{2}G_{H_{2}\left(g\right)}-eU$$ (5)

$$\Delta G_{2}=\Delta G_{O*}-\Delta G_{HO*}+\frac{1}{2}G_{H_{2}\left(g\right)}-eU$$ (6)

$$\Delta G_{3}=\Delta G_{HOO*}-\Delta G_{O*}+\frac{1}{2}G_{H_{2}\left(g\right)}-eU$$ (7)

$$\Delta G_{4}=\Delta G_{O_{2}\left(g\right)}+\Delta G_{*}-\Delta G_{HOO*}+\frac{1}{2}G_{H_{2}\left(g\right)}-eU$$ (8)

## Regulation of Active Site for Promoting OER

### Precious Metals Single Atom Catalyst

**FIGURE 1:**
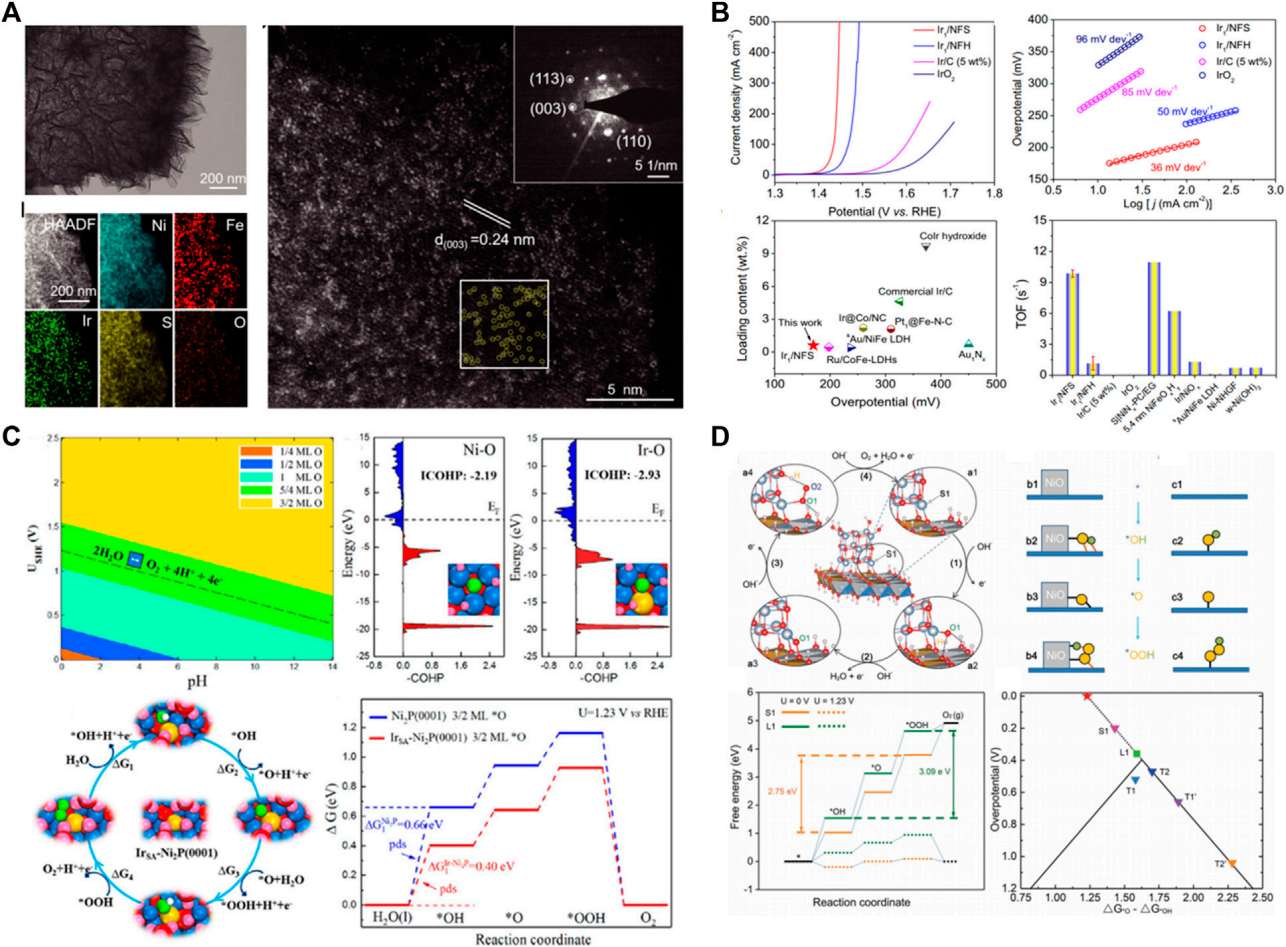
**(A)** Electron microscope analysis of Ir_1_/NFS. **(B)** Electrochemical OER performance of Ir_1_/NFS, Ir_1_/NFH, Ir/C (5 wt%), and IrO_2_ in 1.0 M KOH. **(C)** DFT calculation indicated that the isolated Ir-O-P/Ni-O-P bonding can optimize the adsorption energy of OER intermediate species to accelerate the adsorption/desorption process. **(D)** Structure model and DFT + U calculation results of NiO/NiFe LDH interface.

Precious metals as OER catalysts were reported as early as the 1860s, and the catalytic activity of different precious metals from low to high is Ru, Ir, Pd, Rh, and Pt in acidic electrolyte (Damjanovic et al., 1966; Chen Z. et al., 2021). Under the electric water driving voltage, the surface of precious metals will be transformed into corresponding oxides due to the strong oxidation atmosphere, and the surface will generate an oxide layer. The higher the electrocatalytic activity is, the easier it is to adsorb the intermediate state of reaction and form oxide layer. So, Ru has the highest catalytic activity but poor stability, while Pt requires a high overvoltage to occur OER but has good stability (Danilovic N. et al., 2014). At present, noble metal oxides such as rutile structure RuO_2_ and IrO_2_ exhibit excellent electrocatalytic activity under both acidic and alkaline conditions, and have been regarded as the benchmark of OER electrocatalysts. However, the employment of precious metal significantly aggravates the industrial hydrogen production costs *via* electrochemical water splitting. Single-atom catalysts (SACs) can minimize the utilization of precious metals by maximizing the atom efficiency; meanwhile, the controllable coordination environment and metal support interactions endow SACs as a new paradigm toward designing high-performance OER catalysts. The individual atomic structure of SACs and the interfacial effect between single atom and substrate support can effectively control the electron structure, which endows SACs with suitable and adjustable adsorption energy for reaction species, accelerating the OER kinetic process. Cao et al. (Lei et al., 2022) anchored high distribution density Ir single atom on the nickel-iron sulfide substrate support (Ir/NFS) by a simple two-step electrodeposition (Figure 1A). The obtained Ir/NFS catalyst possesses an excellent OER catalytic performance with a low overpotential of ∼170 mV at 10 mA cm^−2^ and a high turnover frequency of 9.85 s^−1^ at an overpotential of 300 mV in 1 M KOH electrolyte, which is superior to most previously reported OER catalysts, and even commercial IrO_2_ catalysts (Figure 1B). Recently, Ir single atoms were also anchored on Ni_2_P catalyst (Ir_SA_-Ni_2_P) (Wang et al., 2021), the substrate change results in the significant improvement of OER performance, and Ir_SA_-Ni_2_P obtains a record-high OER performance with an overpotential of 149 mV at 10 mA·cm^−2^. Meanwhile, the Ir_SA_-Ni_2_P has a large current density of 154 mA·cm^−2^ at an overpotential of 300 mV, which is almost 28-fold that of the most widely used IrO_2_ catalyst. Gu et al. think that the isolated Ir-O-P/Ni-O-P bonding can optimize the adsorption energy of OER intermediate species to accelerate the adsorption/desorption process (Figure 1C). Furthermore, Cui et al. employ *in situ* cryogenic-photochemical reduction synthesis and DFT method to trace the origin of enhanced water oxidation activity of Ir single atom on NiFe oxyhydroxide substrate (Zheng et al., 2021). The X-ray absorption near edge structure (XANES) spectra results show that the valence of +5.3 for Ir_0.1_/Ni_9_Fe SAC, and the high-oxidation state of Ir possesses excellent OER performance with an overpotential of 183 mV at 10 mA·cm^−2^. The DFT results reveal that the high-oxidation state NiFeIr SAC (+4.8 and +5.6) models can weaker *OH binding and improved *OH and *OOH scaling to obtain a very low overpotential of 0.184 V.

### High-Entropy Alloy Electrocatalyst

High-entropy alloy has been considered as the remarkable nonprecious metal-based OER electrocatalysts due to their adjustable composition, complex surface, enhanced wear resistance, etc. (Li et al., 2021; Park et al., 2022; Han et al., 2022). More importantly, OER is a four-electron/four-proton reaction process and involves the adsorption/desorption of several intermediates (HO*, O*, and HOO*) on the catalyst surface. Numerous studies revealed that the adsorption energy of intermediates has scale relation, such as ΔG(*OOH) = ΔG(*OH)+3.2, the ΔG_*OOH_ can be restricted by ΔG_*OH_, which limit a minimum theoretical overpotential of about 0.37 V, and the adsorption energy of intermediates cannot be optimized independently on a catalyst composed of a single type of site. So the synergistic effect of multi-site was put forward to break or overcome the adsorption-energy scaling relations (Song et al., 2021). Du et al. design the NiO/NiFe LDH interface to introduce additional active sites, and the intermediates can be adsorbed by the interfacial dual-site, so the adsorption energy of each intermediate can be adjusted independently to decrease the overpotential into 0.2 V (Figure 1D) (Gao et al., 2019). As expected, NiO/NiFe LDH possesses a rather high TOF (0.71 s^−1^) and ultralow overpotential (0.2 V at 30 mA cm^−2^). High-entropy alloy has multiple attributes sites to synergistically reduce the multistep process of OER instead of the simple superposition of the performance of each metal ion, so multicomponent alloy generally possesses reinforced electrocatalytic performance compared with that of single-element or bimetallic alloy materials.

Jiang et al. synthesize CoNiCuMnAl)/C nanoparticles using a facile pyrolysis, and the optimized catalyst has low overpotential (215 mV at 10 mA cm^−2^) and Tafel slope (35.6 mV dec^−1^) (Wang et al., 2022). The DFT calculations show that Ni/Co can reduce the barrier potential of rate-determining step (O*→OOH*), and Al sites can promote the electrical conductivity (Feng et al., 2020). Yu et al. introduce Cr into representative NiCoFe system to synthesize (CrFeCoNi)_97_O_3_, and Cr ion can be etched during OER to promote the interfacial reconstruction and amorphization, resulting in an improved OER performance with a low overpotential of 196 mV at 10 mA cm^−2^ and Tafel slope of 29 mV dec^−1^ (Chen Z. J. et al., 2021). Generally, amorphous structure can offer more active sites and thus exhibit better OER performance compared with their crystalline counterparts. Liang et al. employed Mn doping to promote self-construction of active *β*-NiOOH intermediates for NiFeCoMnAl oxide, the *β*-NiOOH with optimized adsorption of *OH and *O intermediates can as active catalytic sites. The prepared NiFeCoMnAl oxide possesses improved OER performance compared with that of the reference sample NiFeCo and NiFeCoAl oxide (Han et al., 2022). Although high-entropy alloys have potential applications in electrocatalytic OER fields, they suffer from various challenges, such as complex synthesis method and the coordination mechanism of multisites.

### Transition Metal Oxide Electrocatalyst

Since the earliest catalysts for electrocatalytic oxygen evolution, nickel, cobalt, iron-based oxides, hydroxides materials have lower catalytic activity than the precious metal iridium ruthenium compound, but their advantages such as lower price and excellent stability are bound to become the focus of research (Zhang et al., 2018; Gao et al., 2021; Wu et al., 2021). Cobalt oxide is at the top of the OER volcano diagram. Meanwhile, Co_3_O_4_ did not change significantly in the process of high potential and OER test by comparing the absorption spectra of Co_3_O_4_ under different voltages, which means that Co_3_O_4_ can maintain a very stable structure under the condition of OER reaction; thus, considerable efforts have been devoted to exploring efficient Co_3_O_4_ electrocatalysts. Co_3_O_4_ belongs to the spinel family, in which Co +2 and Co +3 play different roles in OER process (García-Mota et al., 2012; Bergmann et al., 2015; Lee et al., 2022) Wang et al. (Bo et al., 2005) studied the role of Co +2 and CO +3 ions in the OER process by replacing Co +2 with Zn and Co +3 with Al. The results show that the OER activity of CoAl_2_O_4_ (in the presence of Co +2) is similar to that of Co_3_O_4_ (in the presence of Co+2), but superior to that of ZnCo_2_O_4_ (in the presence of Co +3), which confirms that the bivalent Co+2 plays a dominant role in OER activity.

**FIGURE 2:**
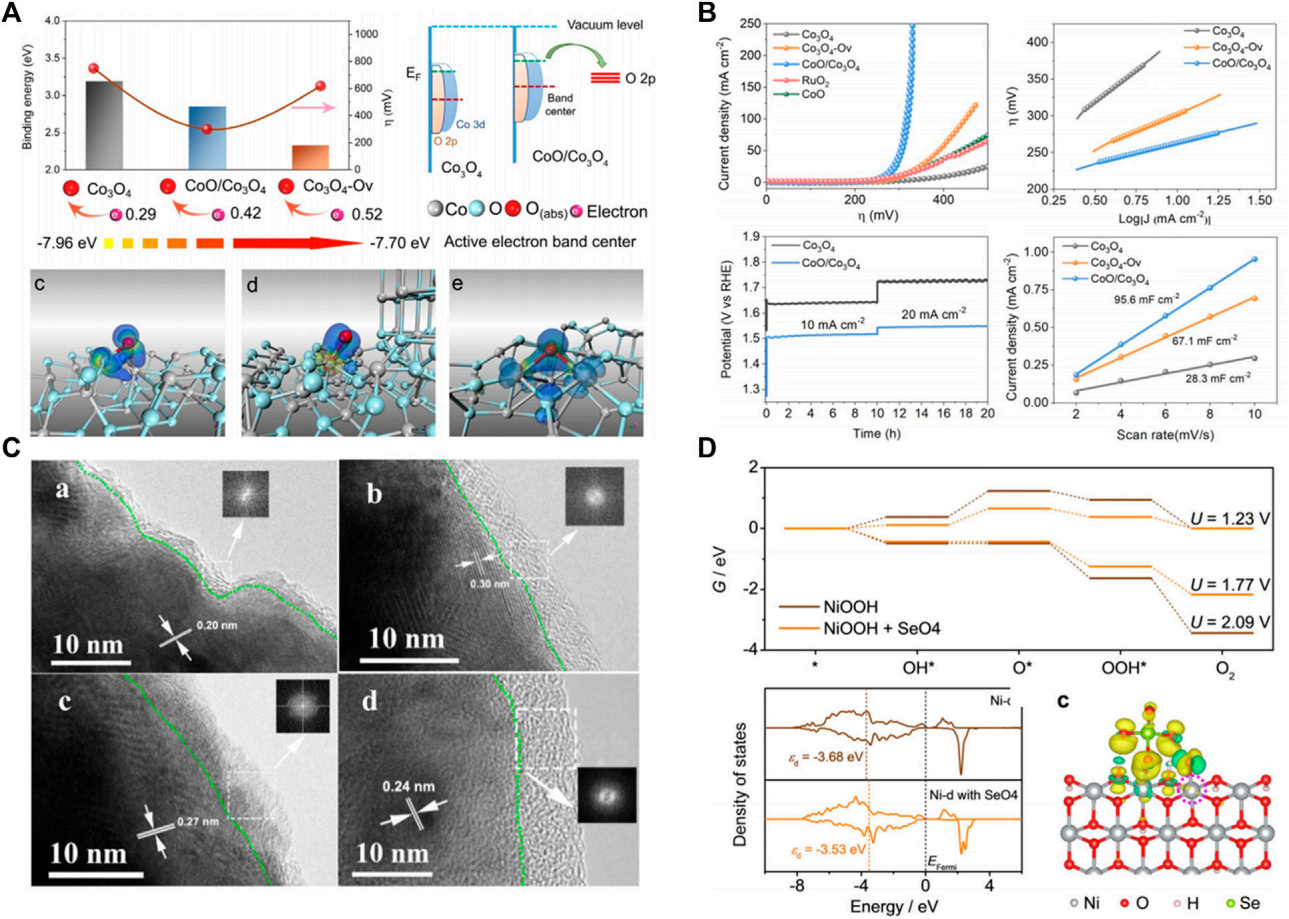
**(A)** DFT calculations of Co_3_O_4_, CoO/Co_3_O_4_, and Co_3_O_4_-Ov. **(B)** Electrochemical catalytic measurement of OER on Co_3_O_4_, Co_3_O_4_-O_v_, CoO/Co_3_O_4_, CoO, and RuO_2_. **(C)** HRTEM images of postcatalysis samples of Ni, Ni_3_Se_2_, NiSe, and NiO. **(D)** DFT calculations of NiOOH and NiOOH + SeO_4_ indicate that the adsorption of SeO_4_ on NiOOH can shift up the d band center, resulting in a stronger bonding with the OER intermediates.

Electronic structure modulation is widely used to regulate the active site so as to optimize the macroscopic catalytic performance of catalysts. Wang’s group (Xu L. et al., 2016) modified the surface of Co_3_O_4_ catalyst by plasma technology. Plasma etching produces more oxygen vacancies on the surface of Co_3_O_4_, which is beneficial to increase the specific surface area of the material and ensure that the Co_3_O_4_ surface has more OER catalytic active sites. In addition, oxygen vacancies introduce impurity energy into the Co_3_O_4_ band gap, which improves the electrical conductivity of the material. Compared with the original Co_3_O_4_, the plasma-sculpted Co_3_O_4_ has a faster OER kinetic process and a lower initial potential. At a voltage of 1.6 V, the current density of the plasmonized Co_3_O_4_ nanosheets was 10 times that of the untreated Co_3_O_4_. Xiao et al. (He et al., 2020) also adopt a novel highly controlled ion irradiation technology to regulate the electronic properties of Co_3_O_4_, and the active electron density can be precisely regulated by the concentration of oxygen vacancy contained Co_3_O_4_ (Co_3_O_4_-Ov) (Figure 2A).The optimized Co_3_O_4_-based catalysts exhibit an excellent overpotential of 260 mV at 10 mA cm^−2^(Figure 2B). Jiang et al. also report that oxygen vacancy not only enhance the adsorption of intermediates, and can also affect the water oxidation path (Figure 2B) (Wang et al., 2017).

### Transition Metal Non-oxides

Although transition metals oxides have been regarded as a potential non-noble OER catalyst in alkaline conditions, their semiconductor property restricted industrial applications. Metal-phase transition metal sulfur (selenium, phosphorus, nitrogen) compounds, due to their good electrical conductivity, can effectively reduce the overpotential caused by impedance, superior to the corresponding semiconductor properties of transition metal oxides, and is also widely used in OER electrode materials. But OER often work in a larger potential, oxygen intermediates (*O, *OH, and * OOH) easily adsorbed on the catalyst surface, causing the transition metal (nitrogen, phosphorus, selenium) compound in the process of OER irreversible oxidation process of surface, the surface gradually phase transition metal oxide or hydroxide (Ji et al., 2020; Xu K. et al., 2016). Xie’s group (Xu K. et al., 2016) systematically compared and analyzed the structural stability of Ni, Ni_3_Se_2_, NiSe, and NiO as oxygen evolution electrodes before and after testing, and found that the surface of the catalyst would be oxidized to form an oxide layer of about 10 nm (Figure 2C). Mabayoje et al. (Mabayoje et al., 2016) also found a similar conclusion that the XRD of NiS after OER test failed to observe obvious diffraction peaks of nickel sulfide and nickel oxide. Furthermore, XPS results indicate that the surface of NiS is transformed into NiO_x_, which means that the surface phase of sulfide is transformed into amorphous oxide under OER operation. Cui et al. also report that CoS_2_, Co_0.5_Fe_0.5_S_2_, and Co_0.37_Ni_0.26_Fe_0.37_S_2_ were converted to CoO, Co_0.5_Fe_0.5_O_2_, Co_0.37_Ni_0.26_Fe_0.37_O_2_, respectively, during cyclic voltammetry by SEM and TEM results (Chen et al., 2015). Although the *in-situ* formed oxides are recognized as the real active species because t the catalyst reaction site mainly comes from the surface, the different transition metals (selenium, phosphorus, nitrogen) possesses distinct OER performance, and the role of nonmetallic elements on OER is still indistinct. Zhong et al. researched the influence of nonmetal S atom in OER of FeMOF, and the results showed that *in situ* formed -SO_3_ groups under OER condition can stabilize *OH or *OOH species by capturing their H^+^, which can break linear relationships between ΔG_OH_ and ΔG_OOH_ to reduce the overpotential. As a result, FeMOFs-SO_3_ shows a low overpotential of 218 mV at a current density of 10 mA cm^−2^. Zhang et al. also report that in-site formative selenate (SeO_4_^2-^) during the OER process can promote the OER activity (Shi et al., 2020). DFT calculations indicate that the adsorption of SeO_4_ on NiOOH can shift up the *d* band center, resulting in a stronger bonding with the OER intermediates (Figure 2D).

## Summary and Perspectives

The OER is a key process involved in energy and environment-related technologies, a great deal of research work has been focused on the development of efficient and stable OER electrocatalysts. Different catalytic systems, such as precious metals single-atom catalyst, high-entropy alloy, transition metals oxides, and transition metal chalcogenide, have unique features and advantages as OER electrocatalysts, and their performance records are constantly being improved by modified active center. On the one hand, by optimizing the electronic structure of the active site, the adsorption energy of the intermediate can reach the fixed point of the volcano diagram. On the other hand, the linear constraint between adsorption energy can be broken through the coordination of multiple sites, so as to obtain excellent catalytic performance.

However, the current OER catalysts suffer from various challenges. 1) The activity-stability of relationship. Stability is a another important parameter of OER for industrialization, most reported catalysts for OER undergo *in situ* electrochemical tuning to form the amorphous oxidation layer, which is the main active species for OER, the faster *in situ* electrochemical transformations can promote the OER activity. So the activity and stability of catalyst have some relationship, which requires the *in situ* characterization techniques and theoretical calculations for a deeper investigate. 2) The structure-activity of relationship. The adsorption energy of reaction intermediate depends on electron structure of catalyst, but the correlation between multiple reaction intermediate (*OH, *O, and *OOH) and electron structure is not identified. And the multisite synergistic effect between metal and metal sites or metal and nonmetal sites has not been deeply revealed. In general, a deeper understanding of OER reaction mechanism is urgently needed to support the design and exploitation of high-performance catalyst in the future.
